# Foodborne Transmission and Clinical Symptoms of Honey Bee Viruses in Ants *Lasius* spp.

**DOI:** 10.3390/v12030321

**Published:** 2020-03-17

**Authors:** Daniel Schläppi, Nor Chejanovsky, Orlando Yañez, Peter Neumann

**Affiliations:** 1 Institute of Bee Health, Vetsuisse Faculty, University of Bern, 3097 Bern, Switzerland; ninar@volcani.agri.gov.il (N.C.); orlando.yanez@vetsuisse.unibe.ch (O.Y.); peter.neumann@vetsuisse.unibe.ch (P.N.); 2 Department of Entomology, Agricultural Research Organization, Volcani Center, 50250 Bet Dagan, Israel; 3 Swiss Bee Research Centre, Agroscope, 3097 Bern, Switzerland

**Keywords:** Acute bee paralysis virus, *Apis mellifera*, clinical symptoms, Deformed wing virus, foodborne virus transmission, *Lasius niger*, *Lasius platythorax*

## Abstract

Emerging infectious diseases are often the products of host shifts, where a pathogen jumps from its original host to a novel species. Viruses in particular cross species barriers frequently. Acute bee paralysis virus (ABPV) and deformed wing virus (DWV) are viruses described in honey bees (*Apis mellifera*) with broad host ranges. Ants scavenging on dead honey bees may get infected with these viruses via foodborne transmission. However, the role of black garden ants, *Lasius niger* and *Lasius platythorax*, as alternative hosts of ABPV and DWV is not known and potential impacts of these viruses have not been addressed yet. In a laboratory feeding experiment, we show that *L. niger* can carry DWV and ABPV. However, negative-sense strand RNA, a token of virus replication, was only detected for ABPV. Therefore, additional *L. niger* colonies were tested for clinical symptoms of ABPV infections. Symptoms were detected at colony (fewer emerging workers) and individual level (impaired locomotion and movement speed). In a field survey, all *L. platythorax* samples carried ABPV, DWV-A and –B, as well as the negative-sense strand RNA of ABPV. These results show that *L. niger* and *L. platythorax* are alternative hosts of ABPV, possibly acting as a biological vector of ABPV and as a mechanical one for DWV. This is the first study showing the impact of honey bee viruses on ants. The common virus infections of ants in the field support possible negative consequences for ecosystem functioning due to host shifts.

## 1. Introduction

The global alarming decline of the entomofauna may yield consequences for the functioning of natural ecosystems and food security [1,2,3,4], because insects provide key ecosystem services, including pollination, natural pest control and decomposition [5]. These ecosystem services are of immeasurable economic value and are critical for human welfare, since they include the provision of food and the regulation of water, among many others [6]. Emerging infectious diseases (EIDs) have been identified as one of the drivers of the recent insect decline [7,8]. EIDs can cause significant impact on human and animal health [9,10,11]. Understanding the transmission, host range and impact of EIDs is crucial for evidence-based mitigation efforts [12].

Host shifts, where pathogens are transmitted from the original host species to a novel one, often precede the emergence of an infectious disease [13,14]. The lack of a shared co-evolutionary history can lead to tremendous effects on novel host populations [15]. In particular, RNA viruses are known to cross species barriers frequently due to error-prone replication enabling fast adaptive changes [16,17]. Therefore, it is not surprising that many RNA viruses are multi-host species [18]. Accordingly, many viruses first described in Western honey bees *Apis mellifera* (henceforth, honey bee viruses) have been identified as multi-host pathogens [19]. Transmission of such RNA viruses between managed honey bees and other species appears to be widespread and may pose a serious threat to wild pollinators [20]. However, the actual transmission pathways as well as the impact, i.e., why certain hosts are infected and others not, remain often poorly understood.

The identified transmission routes for honey bee viruses include horizontal pathways, such as vector-borne transfer by parasitic mites, foodborne-, venereal- and faecal-oral transmission, as well as vertical transmission from the queens to their eggs [21]. Although foodborne transmission seems to be less effective in honey bees, it may nevertheless be a crucial pathway for the infection of other species [22], especially predators and scavengers [23,24]. However, at present, there are little data on the actual impact of honey bee-derived viruses on alternative hosts. Most studies have only reported the mere detection of the viruses with PCR. Even though the detection of negative-sense strands from single-stranded RNA viruses can be used as a token of virus replication, recently consumed viral particles can lead to false positive results in the case of predatory or scavenging insects [24]. Therefore, it appears that infection experiments are required to control the actual virus uptake and enable the detection of clinical symptoms as a clear indication of an overt virus infection [25]. As yet, only a few studies have described the clinical symptoms of honey bee viruses in alternative hosts. Hitherto, the effects of honey bee viruses have mostly been reported in bumblebees *Bombus* spp. (reviewed by Tehel, Brown and Paxton [19]). Pathogenicity in *Bombus* spp. has been shown for acute bee paralysis virus (ABPV), deformed wing virus (DWV), Israeli acute paralysis virus and Kashmir bee virus (KBV). Apart from those, only one study has reported symptoms of DWV in *Vespa crabro* [26]. This appears surprising because DWV has a host range spanning at least 65 species across eight insect orders and three orders of Arachnida [22]. This virus has become ubiquitous in managed honey bees due to efficient vector-borne transmission via ectoparasitic mites *Varroa destructor* [27,28,29]. Clinical symptoms of this single-stranded positive-sense RNA virus, of which at least three distinct genotypes or master variants—type A, B and C—are known so far, include crippled wings, a shortened abdomen and a reduced host lifespan [30]. ABPV is another single-stranded positive-sense RNA virus, which can induce adult mortality, preceded by rapidly progressing paralysis, trembling movements, inability to fly and a gradual darkening and loss of hair at the thorax and abdomen [31].

In a previous study, it has been shown that foodborne transmission is an underlying transmission mechanism, enabling honey bee viruses to infect ants *Myrmica rubra* [24]. Ants are often scavenging in apiaries [32]. Therefore, the consumption of infected food, i.e., virus-infected brood and adults that are often expelled from honey bee colonies as part of their hygienic behaviour and parasitic mites (*Varroa destructor*), major virus vectors, is very likely [33,34]. Ants are potential hosts of honey bee viruses [24,32,35,36,37,38,39], and viral replication has been confirmed for chronic bee paralysis virus in the carpenter ant, *Camponotus vagus* [35], for DWV and Kashmir bee virus in the Argentine ant, Linepithema humile [37,39] and DWV in *Myrmica rubra* [24]. Although these viruses can modify ant immune responses [40], no study has yet experimentally addressed the clinical symptoms of these viruses in ants, either at the individual host or colony level, to confirm that ants are biological hosts. As ants provide essential ecosystem services and play a key role in terrestrial ecosystems [41,42], the potential negative impacts of virus transmission between managed honey bees and ants might pose a threat to ecosystem functioning.

Here, we empirically tested for the first time whether black garden ants, (*Lasius niger*) and *Lasius platythorax,* endemic to Europe [43] are alternative hosts of ABPV and DWV-B using laboratory experiments and a field survey. We initially conducted a feeding experiment to see if either of these viruses are able to infect *L. niger* and how they are distributed within the colonies. Then, another assay was used to investigate clinical symptoms of an ABPV at both individual and colony level. Finally, ants were collected in the field and also diagnosed for virus infections.

## 2. Materials and Methods

### 2.1. Foodborne Transmission of DWV and ABPV

Gynes of *L. niger* (*N* = 20) were collected in Lausanne (August 2013) and Bern (July 2014), Switzerland. Prior and during the experiment, the colonies were kept in nesting tubes (155 mm length, Θ = 14 mm) and fed weekly with collembola and sugar water (33% mass fraction of sugar) ad libitum [24]. A sterile cotton wool ball separated the nesting tubes into two compartments. The rear chamber was filled with sterile water to keep the nests moist. The first cell housed the colony and was closed by another cotton wool plug. The nesting tubes were wrapped in aluminium foil, maintained at room temperature (19–23 °C) and protected from direct sunlight. At the beginning of the experiment (25 August 2014), the colony size ranged from 10 to 200 individuals due to different colony ages. Then, a ten-week feeding regime was launched, long enough for at least one brood development cycle [44]. The treatment colonies received sugar water ad libitum and one honey bee pupae infected with DWV-B and ABPV per week. Since foodborne virus transmission is not very efficient [45], and the overall sample size was rather low (*N* = 20), only two colonies were randomly chosen as controls to increase the chance of detecting a virus infection in the treatments (*N* = 18). The controls were maintained with the previous feeding regime of sugar water and collembola. Three weeks after the last feeding event with virus-infected pupae (24.11.14), nineteen of the twenty colonies were shock-frozen and stored at −80 °C until further processing. From the last colony, only twenty adult workers were collected to keep the colony for further monitoring. The time gap between the last feeding of the virus until the sampling was chosen to minimize the risk of false positive results, since the ant’s gut content should have been emptied at least once in this time window [46,47].

### 2.2. Preparation of Virus-Infected Honey Bee Pupae

ABPV and DWV-B were propagated in microinjected honey bee pupae obtained from two local (Bern-Liebefeld, Switzerland) colonies using standard methods [48]. In brief, we obtained the injection solution from a fresh propagation in red-eyed honey bee pupae injected with a solution containing ABPV and DWV-B and incubated at 34.5 °C, ≥50% relative humidity and darkness for seven days [49]. These bees were then homogenized in PBS buffer (Phosphate Buffered Saline; pH 7.4) and chloroform, followed by centrifugation (15800 rpm for 10 min). The supernatant was isolated, diluted 1:1000 and frozen at −80 °C before use as an injection solution [48]. Freshly collected pupae were then microinjected laterally between the second and third segment of the abdomen with two μL of a solution containing both viruses [48], incubated at 34.5 °C, ≥50% relative humidity and darkness for five days and frozen at −80 °C until feeding or further processing [49]. Presence of the viruses was confirmed using qPCR with virus titers (Log_10_ genomic copies/pupae) in the range of 9.57 ± 1.14 for ABPV and 11.23 ± 0.33 for DWV-B.

### 2.3. Field Survey

On 29 August 2016, 10 hives housing local *A. mellifera* colonies in Bern-Liebefeld (Switzerland) were screened for the presence of ants. Adults (*N* = 10 per hive) and larvae from two hives (each *N* = 3) were sampled, the adults determined to be *L. platythorax* using morphometric characteristics [43] and frozen at −80 °C until further processing.

### 2.4. Clinical Symptoms of ABPV

Based on the results from the feeding experiment, we tested new colonies for clinical symptoms of ABPV with a behavioural assay after a ten-week feeding regime. New colonies were raised from field-caught gynes in nesting tubes (*N* = 16; collected in Bern, Switzerland, 30 July 2016). After overwintering (17 weeks), a foraging arena (135 mm × 68 mm × 32 mm) was attached to the nesting tube (Figure S1). Prior to the experiment, ants were provided weekly with sugar water (40% mass fraction of sugar) ad libitum and drosophila flies (*Drosophila hidey*). Upon initiation of the experiment (July 2017), the colonies were assigned to two feeding regimes in a stratified random way, accounting for colony size. The treatment colonies (*N* = 8) received honey bee pupae microinjected with a solution containing ABPV, while the controls (*N* = 8) received non-injected pupae. As mentioned above, white-eyed honey bee pupae were collected from sealed worker brood frames of local colonies. Half of the pupae were microinjected with 2 μL of a solution containing ABPV. Then, all pupae were incubated at 34.5 °C, ≥50% relative humidity and darkness for five days and then frozen at −80 °C until feeding [48].

Two weeks after the end of the feeding regime, for each colony, five randomly chosen foragers taken from the foraging arena were tested in a behavioural assay. A petri dish (Θ = 90 mm) was used as arena with a reference grid (grid cell: 8.5 mm × 8.5 mm) printed on the bottom and the behaviour was filmed using a camera (Canon EOS 5D Mark IV). For the behavioural assay, individual ants were transferred to the plate and then immediately recorded for 120 sec. The movement of the ants was analysed from slow motions of the videos by quantifying their movement in relation to the reference grid as the number of grid cells passed. If an ant touched two cells at the same time, e.g., while walking along a line, only one was counted. The following measurements were derived from the videos: (I) overall movement—the total number of grid cells passed over 120 sec; (II) time inactive—the amount of time the ants did not move, including standing still or grooming; (III) average speed—the number of grid cells crossed during the time the ants were moving; (IV) initial speed—number of grid cells crossed during the initial 10 sec. The raw data are accessible in Table S1. Three weeks after the last feeding event with honey bee pupae, the colonies were shock frozen and stored at −80 °C. Colony size was assessed again, as well as the weight of the queen and twenty pooled workers. 

### 2.5. RNA Extraction

For the detection and quantification of the viruses, RNA was initially extracted from 10 randomly selected, pooled adult workers and the queens of each colony from the feeding experiment. To further examine virus load and the possibility for vertical transmission in infected colonies, RNA was also extracted from 10 additional workers (*N* = 3 colonies) and eggs (*N* = 1 colony, 5 individual eggs, 3 × 3 eggs pooled, 1 × 10 eggs pooled). To avoid false positives, surface contamination was eliminated with a washing step prior to the extraction [50]. This was accomplished by washing the samples for 1 min in a 2% sodium hypochlorite solution followed by rinsing off with distillate water twice [50,51]. For the survey, we tested ten pooled field-collected samples with ten *L. platythorax* adults each, and two pooled samples, three larvae each. For the behavioural essay, a pooled sample of 40 ants without their abdomens was analysed from each colony. 

The samples were manually crushed with a small mortar in 1.5 mL Eppendorf^®^ tubes (Basel, Switzerland) in TN buffer (100 mM Tris, 100 mM NaCl, pH 7.6). Fifty microlitres of the homogenate were transferred to 1.5 mL Eppendorf^®^ tubes. A NucleoSpin^®^ RNA II kit (Macherey-Nagel, Oensingen, Switzerland) was used for the RNA extraction by following the manufacturer’s recommendations. RNA was eluted in 30 µL of elution buffer and stored at −80 °C [52]. To monitor the efficiency of the RNA purification and cDNA synthesis, we tested for β-actin in the samples from the feeding experiment [53]. The same was achieved for the samples of the behavioural experiment by adding 0.3 ng Ambion™ RNA Control 250 to each sample at the first extraction step [54].

### 2.6. Reverse Transcription

We used the M-MLV RT (Promega, Dübendorf, Switzerland) Kit and followed the manufacturer’s recommendations for the reverse transcription. Template RNA was incubated with 0.75 μL of a random hexamer oligonucleotide (100 μM) and H_2_O in a final volume of 17.75 μL for 5 Minutes at 70 °C in a Thermocycler (Biometra, Analytik Jena, Jena, Germany). Five microlitres of 5× Buffer, 1.125 μL nucleoside triphosphate (dNTP) 10 mM and 1 μL reverse transcriptase (M-MLV) were added to a final reaction volume of 25 μL, which was incubated at 37 °C for 60 min for the cDNA synthesis. The resulting cDNA was diluted 1/5 and stored at −25 °C until further processing.

### 2.7. Real-Time Quantitative PCR

Real-time reverse transcription-quantitative polymerase chain reaction (RT-qPCR) was conducted to estimate viral titers. The samples from the feeding experiment were tested for ABPV and DWV-B, the samples from the field survey for ABPV, DWV-A and -B, and the samples from the behavioural experiment just for ABPV. The RT-qPCR was performed using the KAPA SYBR^®^ FAST Universal qPCR kit (KAPA Biosystems, Wilmington, NC, USA) with 12 μL volumes containing 6 μL KAPA SYBR^®^ green reaction mix, 0.24 μL each of the forward and reverse primers (Table 1), 2.52 μL H_2_O and 3 μL diluted cDNA [48]. On all plates, each sample was run in duplicate for both the targeted virus and the exogenous internal reference. Additionally, two no-template negatives plus a ten-fold serial dilution of purified PCR products that served as standard curves were included [55]. An ECO™ Real-Time PCR machine (Illumina, San Diego, CA, USA) processed the reaction at the following qPCR cycling profile: 3 min incubation at 95 °C and 40 cycles of 3 s at 95 °C for denaturation, 30 s at 57 °C for annealing and extension, and data collection. Following the amplification, a melting curve analysis was used to verify the specificity of the PCR products, by reading the fluorescence at 0.5 °C intervals between 55 and 95 °C., The amplification was followed by a melting curve analysis to verify the specificity of the qPCR products. The analysis was done by reading the fluorescence at 0.5 °C increments from 55 to 95 °C. From the q-PCR output data, the standard curves and the experimental dilution factors viral titers, respectively, estimated viral copies per sample were derived [56]. Virus titers were log-transformed to account for the exponential distribution of the data. Throughout the manuscript, the logarithmic values of the viral titers (Log_10_ genomic copies/sample) are reported. Samples that had no peak or a shifted peak in the melting curve analysis were considered negative and assigned zero viral copies.

### 2.8. Negative-Sense Strand-Specific PCR

To test for viral replication, we performed a negative-sense strand-specific RT-PCR with two consecutive reaction steps, namely tagging and amplification of the RNA. DWV-B and ABPV strand-specific PCR were performed for the samples from the feeding experiment, while the samples from the field survey were also tested for DWV-A and samples from the behavioural experiment were just tested for ABPV. The reaction was processed in a Thermocycler (Biometra, Analytik Jena, Jena, Germany) using a Superscript^®^ III reverse transcriptase (Invitrogen, Carlsbad, CA, USA) following the manufacturer’s recommendations. Superscript^®^ III was used because it supports high temperatures which play a role in the avoidance of false positives [62]. One microliter of tagged forward primer (Table 1), 1 μL dNTP 10 mM, template RNA and H_2_O were incubated for 5 min at 65 °C. Then, 4 μL of 5× Buffer, 2 μL 0.1M DTT and 1 μL M-MLV were added for a final reaction volume of 20 μL and a second incubation at 55 °C for 50 min, followed by 15 min at 70 °C for inactivation. 

The obtained cDNA was purified using a NucleoSpin^®^ Gel and PCR Clean-up kit (Marchery Nagel, Oensingen, Switzerland) and eluted in 30 μL elution buffer. After the purification, the diluted cDNA was amplified according to the previously mentioned conventional PCR with MyTag™ kit (Bioline, London, UK) with a Tag oligonucleotide as forward primer and the corresponding reverse primer (Table 1). As a control for the purification of the cDNA, in which the excess of the forward tagged primers should have been removed, for each sample, a PCR reaction without Tag primer was performed. The thermal cycling profile was 2 min incubation at 95 °C followed by 35 Cycles of 20 s at 95 °C (denaturation), 20 s at 56 °C (annealing) and 30 s at 72 °C (extension), finishing with 2 min of 72 °C. The PCR products were analysed by electrophoresis in a 1.2% agarose gel and visualized under UV light. If a clear band was present at 221 bp for DWV-B, 262 bp for ABPV and the associated control was negative, a sample was considered positive.

### 2.9. Statistical Analyses

All statistical analyses were performed using R version 3.5.1 [63]. To compare worker mortality between the treatments, a Mann–Whitney *U*-test was applied because the data were not normally distributed. The variables overall movement, average speed and initial speed each entered a linear mixed effect model (function lmer, package lme4 [64]) as a response variable. We used the factor treatment as the fixed effect and added colony identity and recording date as random factors to control for repeated measuring of workers from the same colonies and a possible effect of the recording day. Visual inspection of residual plots did not reveal deviations from homoscedasticity or normality. To avoid violations of the model assumptions, we modelled time inactive using a Poisson linear mixed effect model. Thus, the time the ants did not move around was rounded to integers and then entered the model as response variable. Again, treatment was used as the fixed effect and colony identity and recording date were used as random factors. To test for differences between the two treatments with regard to the number of adults and pupae present in the colonies at the beginning of the experiment, the change in the number of adults and pupae until the end of the feeding regime and the weight of queens and workers, data were checked for normal distribution with the Shapiro–Wilk test and homogeneity of variances with the Levene’s test and subsequently analysed with a Student’s *t*-test. To compare the prevalence of ABPV positive queens and pooled worker samples, a two-sided Fisher’s exact test was applied.

## 3. Results

### 3.1. Foodborne Transmission of DWV and ABPV

Queen mortality in the feeding experiment was 10%. One queen from a virus-treated colony and one of the two control queens died during the experiment. The average worker mortality (number of dead workers during the 10-week feeding regime) was 7.85 ± 11.67 (mean ± SD) and did not differ between the treatments (Mann–Whitney *U*-test, W = 17, *p* = 0.94). One colony was kept alive for further monitoring. Thus, the queen was not frozen and a sample size of *N* = 18 (workers) and *N* = 16 (queens) for the virus treatment and *N* = 2 (workers) and *N* = 1 (queen) for the controls remained for subsequent virus analyses.

All samples from the control group (queens and pooled worker samples) were negative for DWV-B. In the virus treatment, 66.7% of the worker samples and 18.75% of the queen samples were tested positive for the DWV-B (virus titers (log mean ± SD): workers—3.21 ± 0.88; queens—4.48 ± 0.9). However, no sample was tested positive for the presence of DWV-B negative-sense strand. Individual workers from three positive colonies were not homogeneously infected with DWV-B: 63.3% of the workers were positive (titers: 3.7 ± 0.86), while the rest was negative. In eggs, only trace amounts of DWV-B were detected in three single eggs, in two of the samples with three pooled eggs and the sample with ten pooled eggs (titers below 3.76).

For ABPV, 94.45% of the worker samples and 87.5% of the queens were positive in the virus-treated colonies (titers: workers—4.5 ± 0.89; queens—6.05 ± 2.68). The worker samples from the two control colonies were negative for ABPV, whereas the surviving control queen was positive, with a viral titer of 6.54. In 16.7% of the worker samples and 50% of the queens from the treatment group and the one control queen, we detected the negative-sense strand of ABPV. Workers analysed individually were 100% tested positive for ABPV (titers: 4.52 ± 1.34). Further, all of the tested eggs were slightly positive for ABPV (titer: 2.35 ± 0.28 per egg based on individual egg samples). Sequencing of the PCR Product (sequence uploaded to GenBank: MT141130) confirmed the identity of ABPV found in ants (GenBank accession: MN565031.1, 100% identity, 100% query cover).

### 3.2. Field Survey

All of the three tested viruses (ABPV, DWV-A and DWV-B) were present in all tested samples, including the larvae. Further, we tested for viral replication, showing that the negative-sense strand of ABPV was present in all samples. However, we did not detect any sign of replication for either strain of DVW-A or -B.

### 3.3. Clinical Symptoms of ABPV

**Figure 1:**
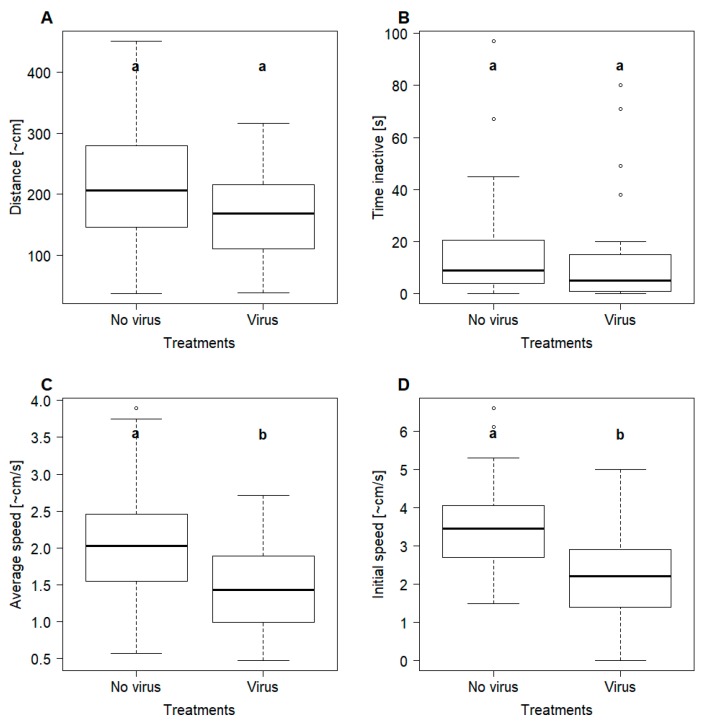
Movement of individual ants (*Lasius niger*) from the two treatments that differed with regard to the feeding regime (controls = no virus, treatment = virus): (**A**) Overall movement of the ants estimated as centimetres crossed during 120 s; (**B**) The amount of time ants were not moving around in the arena; (**C**) Average speed seen over 120 s; (**D**) Distance covered during the first 10 s. A significant difference between the groups (linear mixed effect models, *p* < 0.05) is indicated by different letters (a, b).Furthermore, foragers of the treatment group were observed displaying conspicuous behaviour in the foraging arena. The impaired motions can be described as unnaturally slow movements followed by uncontrolled trembling or twitching motions as well as almost normal movements but with mostly immobile hind extremities (Figure 2).

If seen over the two min observation in the movement arena, there were no significant differences with regard to overall movement (*X*^2^_(1)_ = 2.98, *p* = 0.085; Figure 1A) or the time the ants were not moving (*X*^2^_(1)_ = 2.14, *p* = 0.14; Figure 1B). However, the average speed of the ants from colonies fed with virus-infected pupae was significantly lower than the one from the control treatment (*X*^2^_(1)_ = 5.94, *p* = 0.015; Figure 1C). The difference in speed was even more pronounced for the initial movement speed (i.e., first ten sec), where again the ants from controls were significantly faster than the ones from the treatment group (*X*^2^_(1)_ = 14.66, *p* = 0.0001; Figure 1D).

At the beginning of the experiment, the colonies were assigned to the treatments in a stratified random way to ensure that colonies from the control and the virus treatment did not differ in their colony size (t_13.23_ = 0.81, *p* = 0.43; Figure 3A,B). However, after 13 weeks, there was a significant difference in the number of newly emerged workers, which was higher in the controls (98.88 ± 16.75) compared to the treatment colonies (67.5 ± 22.64; t_12.9_ = 3.15, *p* = 0.008; Figure 3C). Furthermore, while there was no difference in the initial number of pupae present in the colonies (t_13.7_ = 1.15, *p* = 0.88), the virus-fed colonies tend to have fewer pupae (5 ± 5.8) at the end of the experiment compared to the controls (10.5 ± 7.67; t_13.04_ = 1.62, *p* = 0.13; Figure 3D). The bodyweight of queens (controls: 33.11 ± 5.98, virus treatment: 32.56 ± 3.56; t_11.41_ = 0.22, *p* = 0.24) and workers (controls: 20.84 ± 1.54, treatment: 19.68 ± 2.2; t_12.51_ = 1.23, *p* = 0.83) did not differ between the two treatments. The percentages of colonies tested positive for virus presence was significantly different between treatments for pooled workers (virus prevalence—controls: 0%, treatment: 57.14%; *p* = 0.026) but not for queens (virus prevalence—controls: 37.5%, treatment: 87.5%; *p* = 0.12).

## 4. Discussion

Our data show for the first time that ants can display clinical symptoms of honey bee viruses at both an individual and colony level after foodborne transmission. Individual foragers from virus-fed colonies showed impaired locomotion and infected colonies had fewer emerging adults, resulting in smaller colonies. Further, the survey showed that these honey bee viruses can be very common in ants in the field. 

The data support that foodborne virus transmission is indeed an underlying mechanism enabling viruses originally described in honey bees to infect other arthropod predators and scavengers [24,26,35,65,66,67]. Three weeks after the last feeding with virus-infected honey bee pupae, DWV-B was detected in one-third of the pooled worker samples and in 18.75% of the queens. Although we did not detect the DWV negative-sense strand in any sample, our data nevertheless suggest that *L. niger* may act as reservoir host and mechanical vector. *L. platythorax* workers and larvae sampled in the field also carried DWV, both the A- and the B-strain, with no indication of replication. These observations support the wide host range of DWV and two more ant species can be added to the long list of species in which DWV has been detected [22].

ABPV was detected in 94.45% of the pooled worker samples and 87.5% of the queens from colonies fed with infected pupae. Further, the negative-sense strand RNA, indicating ABPV replication, was found in 50% of the queens, including one queen from the controls, and in 16.7% of the pooled worker samples. As all queens were field-collected, it is possible that the queen from the control group had already been infected prior to the experiment. Recently consumed food containing virus particles might lead to false positive results [24]. However, due to the delayed sampling in the feeding experiment (3 weeks), it appears very likely that the gut contents had been cleared out at least once prior to the testing [46,47], thereby probably not biasing our results. Consequently, *L. niger* appears to be an alternative biological host of ABPV and the host range of ABPV may be larger than previously thought [68,69]. Indeed, all of the field samples of *L. platythorax* also carried the negative-sense RNA strand of ABPV, thereby suggesting virus replication and natural infections. These findings are in line with other studies suggesting that ants constitute alternative hosts of viruses previously described in honey bees [24,32,35,36,37,38,39]. Even though it is not clear who is the original host, it appears that ants are novel hosts, which get frequently infected from honey bee reservoirs, because only samples collected in apiaries were tested positive for ABPV and DWV-B, while samples collected further away from apiaries were negative [32].

Differences in infections between workers might be explained either by the age-related division of labour [70], resulting in non-stochastic interaction patterns among individuals [71], or by age and/or differential distribution of protein-rich food. Foragers are most likely the first to get in contact with the virus, as they take up the infected protein source to bring it to the colony. The division into interaction clusters could then limit the spread to some subgroups, restricting the spread of the infection within the colony [71]. On the other hand, due to differential resource-allocation within the colony, queens and larvae receive most proteins and are thus more likely to receive high virus loads [72]. It remains to be investigated how much larvae are affected and if freshly emerged workers are more likely to become infected. Further, the data also suggest possible vertical transmission because both viruses were found in eggs of *L. niger*, albeit at very low titers. Vertical transmission of DWV has been shown in honey bees [25] and the virus appears to adhere to the surface of eggs rather than being transmitted within the eggs [73]. Since the eggs in our experiment were surface-sterilized before virus analyses, handling by infected nestmates can be excluded and it appears that ant eggs can contain some viruses intracellularly via transovarial infection.

**Figure 2:**
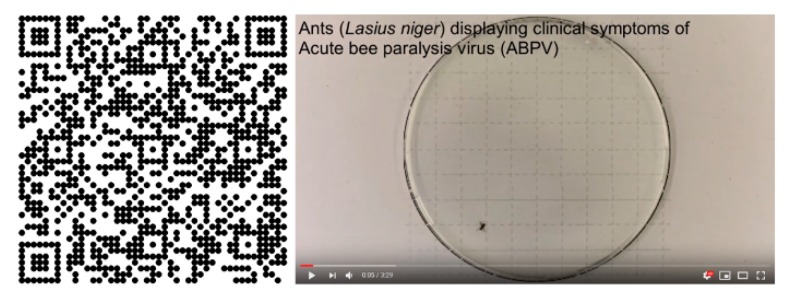
QR code to access the video showing ants (*Lasius niger*) displaying clinical symptoms of Acute bee paralysis virus (ABPV) after experimental feeding with infected honey bee pupae (URL: https://www.youtube.com/watch?v=3bE_3Gl1Pp4&feature=youtu.be).

**Figure 3:**
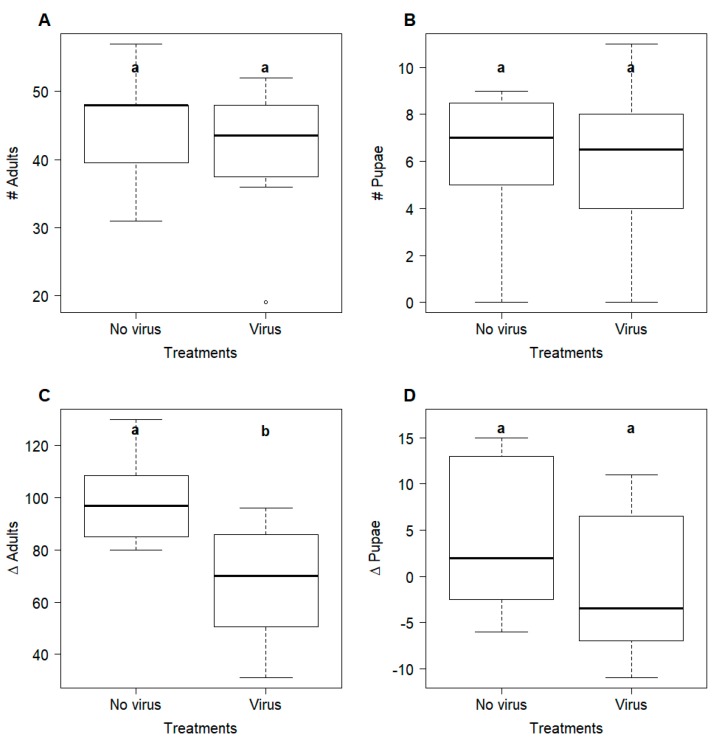
Colony size for the two treatments that differed with regard to the 10 week feeding regime (controls = no virus (*N* = 8), treatment = virus (*N* = 8) measured as the number of *Lasius niger* workers (**A**) and pupae (**B**) upon initiation of the experiment (July, 2016) and the change in the number of workers (**C**) and pupae (**D**) after 13 weeks. A significant difference between the groups (Student’s *t*-test, *p* < 0.05) is indicated by different letters (a, b).

The detection of clinical symptoms of a virus is a clear indication for an overt infection [25]. Therefore, our data show that *L. niger* is a biological host of ABPV, as clinical symptoms were detected at both individual as well as colony level. Infected ants were moving significantly slower (average speed and initial speed) compared to the controls. Additionally, shaky and uncontrolled movements were only detected in individuals from treated colonies (Figure 2). It seems evident that such impaired movement constitutes a clear disadvantage for any inter- and intraspecific conflicts as well as foraging [69]. Overall, the observed clinical symptoms at the individual ant level are very similar to the ones described from honey bees [31].

Similar to our observations of smaller colony sizes of infected *L. niger* colonies, ABPV infections in honey bees can result in a sharp decline of the adult bee population [31]. Since adult mortality was not observed, removal/cannibalism of diseased brood [74,75], resulting in fewer emerging adults, underlies the observed smaller sizes of infected colonies. This would be analogous to the Solenopsis invicta virus 1 (SINV-1), which can also cause increased larval mortality and a loss of body weight in queens [76]. However, our data do not suggest any effect of ABPV on bodyweight, either for queens or workers, which may due to differences between viruses and/or hosts. The three ABPV-positive control queens could be explained either by low background level of viruses in the honey bee pupae food or, alternatively, but not mutually exclusively, by naturally occurring virus infections in the field-collected queens. 

Since larger workforces are more efficient in exploiting and dominating resources [77,78], differences in colony size are likely to increase over time. After eight weeks, the control colonies were already 37.7% larger on average than the colonies fed with virus-infected honey bee pupae. The size of the workforce directly affects colony organisation and productivity, as well as constituting an advantage in interspecific conflicts [79,80,81,82]. Further, larger colonies can be expected to have a higher fitness, as the onset of the reproductive stage is earlier and more gynes and males are being produced [83,84,85,86]. Hence, the observed effects from virus host shifts are potentially of high relevance for colony fitness in the field.

## 5. Conclusions

Ants are not only reservoirs and mechanical vectors, but can also be biological hosts of honey bee viruses. Since foraging ants regularly consume honey bees or other possibly infected food sources, such as *V. destructor* mites [34], foodborne infections and clinical symptoms are likely to occur in the field, as supported by the field survey. Impaired movement capabilities and colony size are both relevant factors for fitness in ants, suggesting that these shifts of honey bee viruses can contribute to the weakening of colonies. This is the first study shedding light on the impact of the two honey bee viruses on ants. There is considerable potential for virus transmission between managed honey bees and ants, with possible consequences for ecosystem functioning and the provision of valuable ecosystem services, due to the important role ubiquitous ants play in terrestrial ecosystems. Understanding of the impact of viruses on ants as alternative hosts might help to find appropriate measures to protect biodiversity and counteract virus-driven insect decline. 

## Figures and Tables

**Table 1:** Primers used for the relative virus quantification and the detection of negative-sense strand RNA of viruses in ants. Exp. = Experiment; 1 – Feeding experiment; 2 – Field survey; 3 – Behavioral assay.

Assay	Target	Primer	Sequence (5′–3′)	[bp]	Ref	Exp
qPCR	DWV-A	DWV F8668	TTCATTAAAGCCACCTGGAACATC	136	[57]	2
		DWV B8757	TTTCCTCATTAACTGTGTCGTTGA			
	DWV-B	VDV-1 F1409	GCCCTGTTCAAGAACATG	413	[58]	1
		DWV B1806	CTTTTCTAATTCAACTTCACC			
	DWV-B	VDV F2	TATCTTCATTAAAACCGCCAGGCT	139	[59]	2
		VDV R2a	CTTCCTCATTAACTGAGTTGTTGTC			
	ABPV	ABPV F6548	TCATACCTGCCGATCAAG	197	[60]	1,2,3
		ABPV B6707	CTGAATAATACTGTGCGTATC			
	Β-actin	Am-actin2-qF	CGTGCCGATAGTATTCTTG	271	[60]	1
		Am-actin2-qF	CTTCGTCACCAACATAGG			
	RNA 250	RNA 250-F	TGGTGCCTGGGCGGTAAAG	227	[54]	3
		RNA 250-R	TGCGGGGACTCACTGGCTG			
Negative sense strand specific PCR	TAG	tag	AGCCTGCGCACCGTGG	-	[61]	1,2,3
	DWV-A	DWV 3Ftag	AGCCTGCGCACCGTGG –GGATGTTATCTCCTGCGTGGAA	221	[24]	2
		DWV 4R1	TGTCGAAACGGTATGGTAAACT	221		
	DWV-B	VDV 3Ftag	AGCCTGCGCACCGTGG–GGATGTTATCTTTTGAGAGGGA	221	This study	1,2
		VDV 4R1	TGTCGGAATGGAATCGTAAATT	221		
	ABPV	ABPV F4868tag	AGCCTGCGCACCGTGG–CAAAACCCGCTATCTTGAGG	262	This study	1,2,3
		ABPV B5114	CCATGGAAAACCTGGTGAAC	262		

## Supplementary Materials

The following are available online at https://www.mdpi.com/1999-4915/12/3/321/s1, Figure S1: Experimental cage with nesting tube and foraging arena, Table S1: Raw data of the behavioural assay.
