# Supplementary material for: Foodborne Transmission and Clinical Symptoms of Honey Bee Viruses in Ants Lasius spp
Source: Viruses. 2020 Mar 17;12(3):321. doi: 10.3390/v12030321 (PMC7150850; doi:10.3390/v12030321)
Supplement: Supplementary file 1 [file viruses-12-00321-s001.pdf]

Supplementary Materials

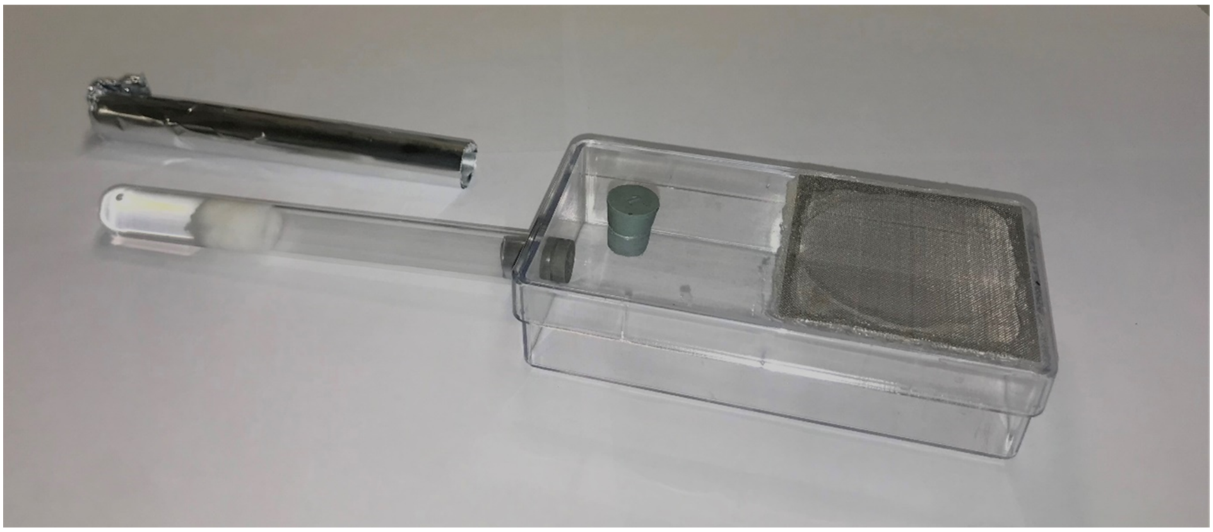

Figure S1: Experimental cages consisting of a foraging arena (135 mm × 68 mm × 32 mm) attached to a nesting tube (155 mm length, Θ = 14 mm). A cotton wool ball separated the nesting tubes into two compartments, the front one housed the colonies and the rear one was filled with water.

Table S1 Raw data of the behavioural assay

colony_id	treatment	time	ant	date	start_time	end_time	active_time	inactive_time	initial_speed	average_speed	overall_movement
1	virus	13:45:00	1	28.09.2018	8.333	128.333	118.496	1.504	25	1.15615717	137
1	virus	15:40:00	2	03.10.2017	6.988	126.988	104.488	15.512	12	0.775208636	81
1	virus	12:51:00	3	06.10.2017	4.178	124.178	105.644	14.356	36	2.167657415	229
1	virus	14:06:00	4	09.10.2017	6.362	126.362	120	0	20	1.633333333	196
1	virus	14:09:00	5	11.10.2017	3.645	123.645	116.57	3.43	22	1.655657545	193
2	control	13:35:00	1	02.10.2017	16.709	136.709	52.755	67.245	34	1.857643825	98
2	control	11:30:00	2	04.10.2017	7.585	127.585	104.344	15.656	32	1.197960592	125
2	control	16:41:00	3	05.10.2017	12.189	132.189	120	0	48	2.775	333
2	control	16:40:00	4	09.10.2017	6.083	126.083	118.694	1.306	40	2.266331912	269
2	control	12:27:00	5	11.10.2017	3.62	123.62	112.027	7.973	33	2.517250306	282
3	control	10:42:00	1	03.10.2017	6.042	126.042	79.856	40.144	40	2.116309357	169
3	control	11:23:00	2	04.10.2017	3.929	123.929	104.858	15.142	29	2.145759026	225
3	control	16:29:00	3	05.10.2017	4.824	124.824	119.19	0.81	27	2.256900747	269
3	control	17:11:00	4	09.10.2017	7.775	127.775	111.238	8.762	15	0.566353225	63
3	control	17:34:00	5	10.10.2017	4.942	124.942	113.923	6.077	24	1.509791701	172
4	virus	15:28:00	1	02.10.2017	21.053	141.053	71.095	48.905	19	0.787678458	56
4	virus	15:23:00	2	03.10.2017	6.521	126.521	119.334	0.666	14	0.988821292	118
4	virus	14:44:00	3	05.10.2017	6.726	126.726	117.838	2.162	16	1.366282523	161
4	virus	11:52:00	4	10.10.2017	9.592	129.592	112.228	7.772	9	0.784118045	88
4	virus	12:50:00	5	11.10.2017	10.092	130.092	81.695	38.305	10	0.465144746	38
5	virus	14:25:00	1	02.10.2017	10.163	130.163	105.181	14.819	42	2.709614854	285
5	virus	16:35:00	2	04.10.2017	11.227	131.227	111.381	8.619	12	1.607096363	179
5	virus	16:33:00	3	05.10.2017	9.567	129.567	111.945	8.055	36	2.349367993	263
5	virus	14:39:00	4	09.10.2017	4.702	124.702	120	0	20	1.675	201
5	virus	16:58:00	5	10.10.2017	4.57	124.57	104.68	15.32	22	1.891478793	198
6	control	15:12:00	1	02.10.2017	10.586	130.586	99.991	20.009	41	1.530137712	153
6	control	12:10:00	2	04.10.2017	7.006	127.006	96.814	23.186	45	1.084553887	105
6	control	14:24:00	3	05.10.2017	9.553	129.553	96.952	23.048	41	1.887531975	183
6	control	11:42:00	4	10.10.2017	7.438	127.438	110.868	9.132	40	1.794927301	199
6	control	12:54:00	5	11.10.2017	4.546	124.546	75.008	44.992	16	0.666595563	50
7	control	13:41:00	1	02.10.2017	7.408	127.408	120	0	35	1.558333333	187
7	control	14:46:00	2	04.10.2017	2.714	122.714	114.761	5.239	49	2.70126611	310
7	control	17:01:00	3	05.10.2017	4.776	124.776	112.962	7.038	61	3.75347462	424
7	control	16:49:00	4	09.10.2017	13.407	133.407	120	0	39	3.2	384
7	control	13:17:00	5	11.10.2017	9.369	129.369	115.719	4.281	66	3.897372082	451
8	virus	15:17:00	1	02.10.2017	0	120	116.299	3.701	8	1.556333244	181
8	virus	15:11:00	2	04.10.2017	4.237	124.237	114.526	5.474	27	2.121788939	243
8	virus	17:14:00	3	05.10.2017	8.446	128.446	114.538	5.462	50	2.610487349	299
8	virus	14:51:00	4	09.10.2017	6.922	126.922	117.96	2.04	44	2.678874195	316
